# Result of the Late Microscopic Observations upon Syphilitic Blood

**Published:** 1872-08-01

**Authors:** Edmund Andrews

**Affiliations:** Professor of Surgery in Chicago Medical College


### RESULT OF THE LATE MICROSCOPIC OBSERVATIONS UPON SYPHILITIC BLOOD.

BY EDMUND ANDREWS, M. D.,

*Professor of Surgery in Chicago Medical College.*

Some years ago, Dr. Saulsbury, of Cleveland, Ohio, gave out that he had discovered certain microscopic corpuscles in the blood of syphilitic patients, which he believed to be peculiar to that disease, and the cause of its symptoms. But little discussion or examination was elicited by this announcement, and the supposed discovery fell stillborn on the world. During the present year, Dr. Lostorfer of Vienna, read a paper before the Vienna Medical Society, in which he announced a similar discovery, but without giving Dr. Saulsbury, so far as I can learn, any credit for his priority. Probably the whole American discussion of the subject was unknown to the surgeons of Vienna. Professors Stricker and Hebra, both eminent men, at once gave in their adhesion to Dr. Lostorfer's views, and some Americans studying in Vienna, enthusiastically reported the discovery to one or two of our Atlantic medical journals, thus launching it a second time before the American profession.

Dr. Lostorfer's observations were made as follows: A very small drop of the blood was placed on a slide and covered in the usual manner with a thin slip of glass. The whole was kept for a day or two in a warm, moist place. If such a slide is examined from time to time with a No. 10 Hartnack's immersion lens, it will be found to present numerous minute vesicles in rapid motion, which gradually acquire a considerable size, sometimes nearly equalling the blood corpuscles. Dr. Lostorfer thought he saw signs of reproduction by germination.

At this stage of the discussion the distinguished Dr. Kobner took up the matter and spent three weeks in a careful series of experiments. Lostorfer had claimed that these vesicles were found only in syphilitic blood, and that the disease could be diagnosed by their means, but Kobner found them in blood from persons affected with lupus, eczema, acne, small-pox, and also in the blood of persons perfectly free from all disease. He found that by placing the slide in a warm moist receiver, he could cause the vesicles to appear within two hours, and by numerous experiments showed with apparent conclusiveness that they had nothing whatever to do with syphilis. This result therefore deprives the discovery of the corpuscles of all value as a means of diagnosing syphilis, and assigns to Dr. Saulsbury the doubtful honor of priority in making a blunder.

